# Supplementary material for: Guanidines Conjugated with Cell-Penetrating Peptides: A New Approach for the Development of Antileishmanial Molecules
Source: Molecules. 2025 Jan 10;30(2):264. doi: 10.3390/molecules30020264 (PMC11768059; doi:10.3390/molecules30020264)
Supplement: Supplementary file 1 [file molecules-30-00264-s001.zip › molecules-3371948-supplementary.pdf]

Supplementary Materials

Guanidines Conjugated with Cell-Penetrating Peptides: A New Approach for the Development of Antileishmanial Molecules

João Victor Marcelino de Souza [1,†], Natalia C. S. Costa [2,†], Maria C. O. Arruda Brasil [1], Luana Ribeiro dos Anjos [3], Renata Priscila Barros de Menezes [4], Eduardo Henrique Zampieri [3], Jhonatan Santos de Lima [2], Angela Maria Arenas Velasquez [2], Luciana Scotti [4], Marcus Tullius Scotti [4], Marcia A. S. Graminha [2], Eduardo R. Pérez Gonzalez [3,*] and Eduardo Maffud Cilli [1,*]

[1] Department of Biochemistry and Organic Chemistry, Institute of Chemistry, São Paulo State University (UNESP), Araraquara 14800-060, SP, Brazil
[2] School of Pharmaceutical Sciences, São Paulo State University (UNESP), Araraquara 14800-903, SP, Brazil; natalia.costa@unesp.br (N.C.S.C.)
[3] Fine Organic Chemistry Lab, School of Sciences and Technology, São Paulo State University (UNESP),
Presidente Prudente 19060-080, SP, Brazil; luana.anjos@unesp.br (L.R.d.A.)
[4] Natural Products and Synthetic Bioactives Postgraduation Program—Federal Paraiba University (UFPB), João Pessoa 58051-900, PB, Brazil
* Correspondence: eduardo.gonzalez@unesp.br (E.R.P.G.); eduardo.cilli@unesp.br (E.M.C.)
† These authors contributed equally to this work.

Summary

Figures

Figure S1. Representation of the interactions of TAT, GVL1, GVL1-TAT, and Amp B with the residues of the cysteine protease active site from Leishmania.

GVL1

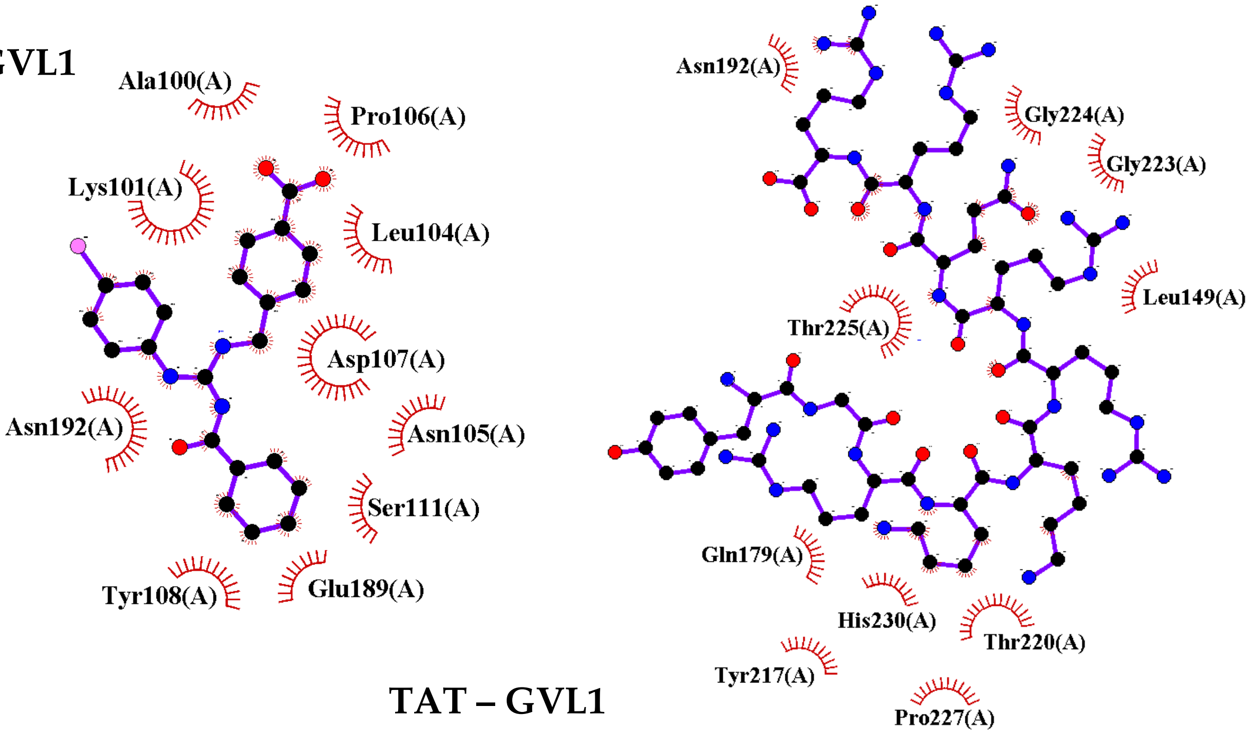

TAT – GVL1

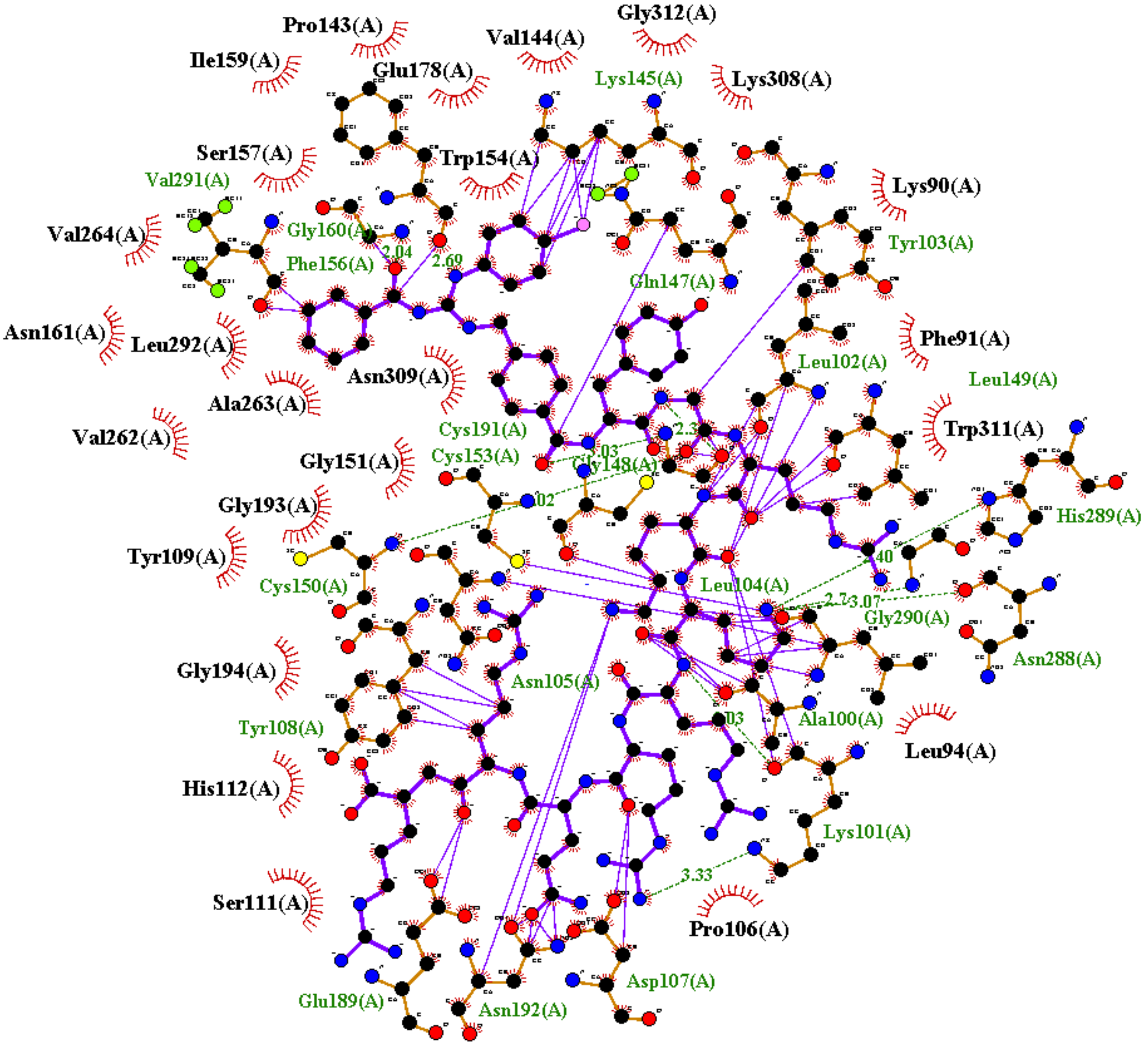

Amphotericin B

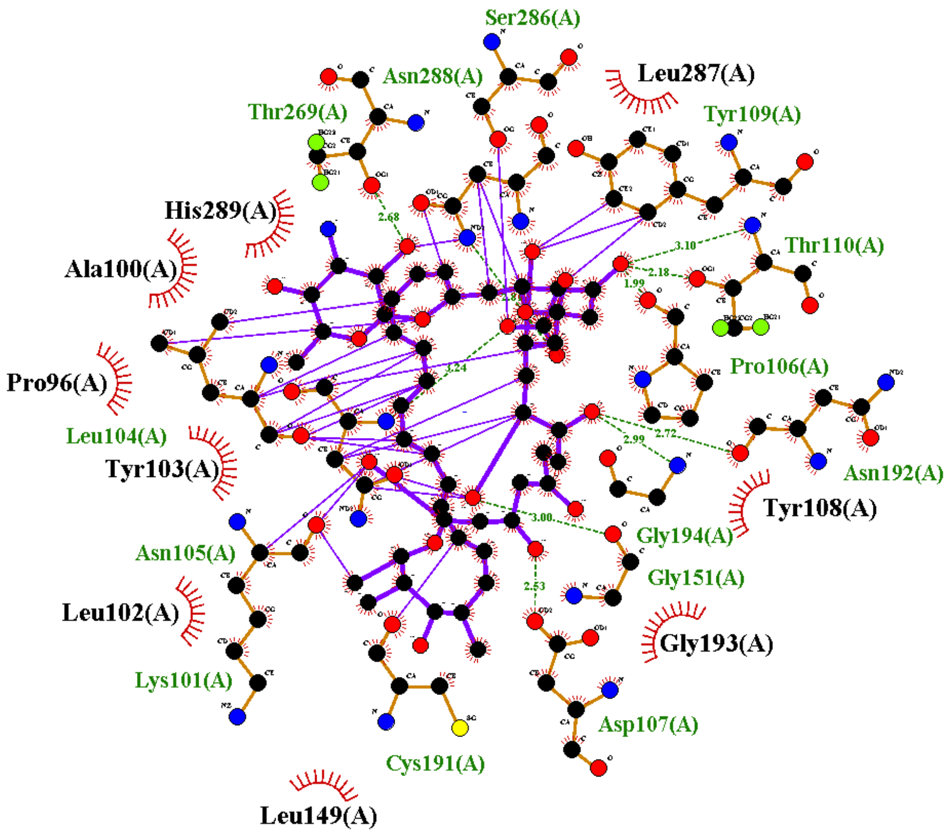

Legend

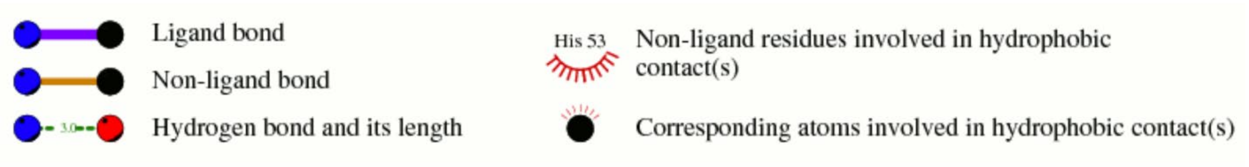

Figure S1: Representation of the interactions of TAT, GVL1, GVL1-TAT, and Amp B with the residues of the cysteine protease active site from *Leishmania*.
